# Double Burden of Malnutrition among Female Adolescent Students in Bahir Dar City, Amhara, Ethiopia

**DOI:** 10.1155/2020/6249524

**Published:** 2020-08-15

**Authors:** Wubet Taklual, Sewunet Baye, Maru Mekie, Tesfaye Andualem

**Affiliations:** ^1^Department of Public Health, College of Health Sciences, Debre Tabor University, Debre Tabor, Ethiopia; ^2^College of Medicine and Health Sciences, Bahir Dar University, Bahir Dar, Ethiopia; ^3^Department of Midwifery, College of Health Sciences, Debre Tabor University, Debre Tabor, Ethiopia; ^4^Department of Medical Laboratory, College of Health Sciences, Debre Tabor University, Debre Tabor, Ethiopia

## Abstract

**Background:**

Globally, nearly a third of the population suffers from at least one form of malnutrition. Both over- and undernutrition are a growing concern in developing countries particularly among female adolescents. This study was aimed at assessing nutritional status and associated factors among female adolescents in secondary schools of Bahir Dar City, Amhara, Ethiopia, 2019.

**Methods:**

A school-based cross-sectional study was conducted in secondary schools of Bahir Dar City among 682 female adolescent students in 2019. A simple random sampling technique with proportional allocation was used to select study participants. Data were collected using a self-administered questionnaire. Data were entered into Epi Info version 7.1 and analyzed by SPSS version 21. Underweight and overweight statuses of the participants were determined by using the WHO cutoff point. Bivariable and multivariable logistic regressions were used to identify the significance of association at a 95% confidence interval. *P* value < 0.05 was used to declare statistical significance.

**Results:**

In this study, the prevalence of underweight, overweight, and obesity was 15%, 8.4%, and 4.7%, respectively. Female adolescents found between age groups of 14-16.5 years old (AOR: 1.7, 95% CI: 1.03-2.69), family size ≥ 4 (AOR: 2.8, 95% CI: 1.05-4.99), participants who did not eat meat once per week (AOR: 1.6, 95% CI: 1.90-2.82), and no onset of menarche (AOR: 4.4, 95% CI: 1.21-15.75) were found to be more likely underweight. In addition, adolescents with family monthly income above 6500 Ethiopian birr (AOR: 12.7, 95% CI: 2.47-65.62), who ate meat two times and more per week (AOR: 2.07, 95% CI: 1.47-9.14), and who ate fruit at least once a week (AOR: 0.20, 95% CI: 0.05-0.78) were more likely to be overweight compared with counterparts. *Conclusion and Recommendation*. The prevalence of underweight and overweight was found to be high. Design evidence-based adolescent nutritional intervention shall be emphasized by the government and other concerned bodies to avert the dual burden of malnutrition.

## 1. Background

Nutrition is recognized as a key determinant of health and well-being and a contributor to human capital development [1]. Poor nutrition starts before birth, continues into adolescence and adult life, and can span generations [2]. Body size during adolescence can be used as a proxy indicator for nutritional status, with overnutrition manifesting as overweight and obesity, while undernutrition can manifest as stunting and/or wasting. Adolescence is a period of rapid physiological, sexual, neurological, and behavioral changes which lays a foundation to adult role and responsibility [3]. Twenty percent of final adult height and 50% of adult weight are attained during the period of adolescence since it is the 2^nd^ period of rapid growth next to the first one year following birth [4, 5]. Investment in adolescent nutrition could have economic and social benefits including saving health care costs, increased intellectual capacity, and adult productivity [6–8].

Globally, nearly one in three persons suffers from at least one form of malnutrition. Rapid nutrition and demographic transition resulted in change in living condition and dietary habit leading to a coexistence of over- and undernutrition [9]. The double burden of malnutrition, both underweight due to undernutrition and overweight due to overnutrition, are a growing concern among female adolescents in developing countries [8, 10–12]. A study conducted among school adolescents in Adama City, Central Ethiopia, indicated that 21.3% and 3.3% of adolescents were under and overweight, respectively [13]. Another study conducted in South Africa about predictors of weight status and obesity disclosed that combined overweight and obesity were found to be higher among female adolescents (15%) compared with male counterparts (4%) [8]. Malnutrition is a multifaceted problem influenced by biological, environmental, social, and behavioral factors [9]. Evidence from different literature indicated that poor dietary diversity score, place of residence, parental level of education, and life styles were predictors of malnutrition among female adolescents [12, 14].

Although malnutrition is a prevailing major public health problem [1], adolescent malnutrition is not well studied particularly in low- and middle-income countries. Little information is available in the Ethiopian context and in Bahir Dar in particular. This study was aimed at assessing malnutrition and its associated factors among female school adolescents in Bahir Dar City, Amhara Reginal State, Ethiopia, which might provide evidence for program implementers and policy makers for decision-making purposes.

## 2. Methods

### 2.1. Study Area, Design, and Population

A school-based cross-sectional study was conducted among female secondary school students in Bahir Dar City, Amhara, Ethiopia, from January to May 2019. In the city, there were 11 high schools (6 governmental and 5 private secondary schools) running a total of 16,644 students at the time of the study. Of these, 8364 of them were females. All secondary school female students in Bahir Dar City were the source population. While all female students in the selected schools were the study population, students who were not attending their education during data collection period, apparent physical deformity (like kyphoscoliosis), cognitive limitation, and those below 10 and above 19 years old were excluded from this study.

### 2.2. Sample Size Determination and Sampling Procedure

The sample size was determined by using a single population proportion formula with the prevalence of underweight 28% and overweight 5.2% [15]. Maximum prevalence was used to calculate the samples size. Other assumptions were 95% confidence (*Z* = 1.96), 5% margin error (*d*), design effect of 2, and nonresponse rate of 10% giving a final sample size of 622. Multistage random sampling technique was used. From a total of 11 secondary schools in Bahir Dar City, three secondary schools (2 governmental and 1 private) were selected using a simple random sampling technique after stratifying secondary schools as governmental and private. The number of schools selected for this study was determined by taking 20% from the total secondary schools as a rule of thumb. Students' rosters from each school were used as the sampling frame to select the study participants using a simple random sampling technique after proportional allocation of samples. Participants were selected by lottery method in each school. Hence, a total of 682 (583 from governmental and 99 from private schools) were selected to participate in this study.

### 2.3. Data Collection Procedure

A pretested structured questionnaire was used to collect the data through a self-administered method. The participants gave their response in a private setting after obtaining the consent from parents and participants. The questionnaire first prepared in English and was then translated into the local language Amharic and back into English by language experts to check consistency.

### 2.4. Study Variables

In this study, the dependent variables were underweight and overweight, measured based on WHO criteria, whereas the independent variables were sociodemographic characteristics of the respondents and parents (age of the respondent, type of school, level of grade, marital status of the respondents, family size, religion, ethnicity, sex of head of the household, occupational status of the father, occupational status of the mother, educational level of the father, educational level of the mother, monthly family income, and means of transport), diet-related information (staple diet, meals per day, vegetable intake per day, fruit intake per week, meat intake per week, snack intake per day, hunger in the last 30 days due to shortage of food, and additional food consume), behavior, life style, and health status (hard physical activities per week, moderate physical activities, sport done (gymnastic, swimming), time taken to school, alcohol drinking habit, cigarette smoking habit, availability of latrine, any illness in the past 2 weeks, and sources of drinking water).

### 2.5. Variable Measurements

Nutritional status of female adolescents (both underweight and overweight) was assessed using anthropometric measurement. A two-day training was given for 6 health officers and 2 human nutrition professionals about the procedures of the data collection instrument and measurements. Weight was measured by a calibrated and portable personal weight scale. During measurement, any heavy clothes, shoes, socks, and items from their pockets were removed from participants. Before measurement, the scale was zeroed, and the study participants stand in the centre without any support until the result is recorded. Zero adjustment on the scale was also made before taking the next measurement [16, 17]. Height was measured using a calibrated stadiometer with head board. During height measurement, the data collector wiped to clean the stadiometer and explained to the study participant about the measurement. Participants were asked to take off their shoes, wear light clothes, and stand with their back to the stadiometer and look forward directly with their arms hanging loosely at their sides. The back of their feet, calves, buttocks, shoulders, and the back of their head were made to be in contact with the stadiometer. Finally, the participants were asked to take a deep breath and hold [18, 19]. All measurements were taken to the nearest 0.1 kg and 0.1 cm for weight and height measurements, respectively. One measurement was taken by two data collectors and a third measurement was taken if the two differed by ≥0.5 kg and ≥0.5 cm for the weight and height, respectively [17, 18].

BMI-for-age *Z*-score values were used to determine the nutritional status of the participants by using the WHO Anthro Plus software. Based on the *Z*-score value, obesity is defined as greater than +2SD, overweight is greater than +1SD, normal weight is between less than +1SD and greater than -2SD, and underweight is less than -2SD [20]. In this study, adolescence is age groups between 10 and 19years old, secondary school is from grades 9 to 12, and physical activity is a total of one hour per day of moderate- to high-intensity physical activities.

### 2.6. Data Processing and Analysis

Data were entered and coded by Epi Info version 7.1 software. The data cleaning and analysis were performed by SPSS version 21 software. Descriptive and analytical statistics were computed. Bivariable and multivariable logistic regressions were conducted to identify the predictors of undernutrition and overnutrition among female adolescent students. *P* value < 0.2 was used to select candidate variables for multivariable analysis. *P* value < 0.05 with a 95% CI was used to decide statistically significant association.

### 2.7. Ethical Clearance

Ethical clearance was obtained from the Research Review Committee of the College of Medicine and Health Sciences, Bahir Dar University. The permission letter was taken from Amhara Regional State Education Bureau, Bahir Dar City Education Department, and respective secondary schools in the city. Informed written consent was received from parents and each study participant. Confidentiality of the information was also maintained.

## 3. Results

### 3.1. Sociodemographic Characteristics of the Respondents

A total of 682 female adolescent secondary school students were included in this study with a response rate of 100%. The mean ages of the respondents were 17.2 ± 1.4 years old. Most of the participants, 617 (90.5%) and 668 (97.9%), were single and Amhara in ethnicity, respectively. The majority, 432 (63.5%), of the study participants reported living in a household with 4-6 family members. Only a third of participants reported to use vehicle as a means of transport on a daily basis (Table 1).

### 3.2. Nutrition- and Diet-Related Characteristics of the Study Participants

Majority of the respondents, 667 (97.8%), used teff as a staple diet. Around two-thirds, 432 (63.3%), of them consume three meals per day. Nearly half of the study participants reported that they consume vegetables and fruits once a day and once a week, respectively. More than half, 387 (56.7%), of them did not eat snacks. Most of the participants, 585 (85.8%), did not face hunger related to shortage of food in the last one month (Table 2).

### 3.3. Behavior and Life Style

More than half, 385 (56.0%), of the study participants spent their time by doing moderate activities. A third of them spent their time by doing hard physical activities. The majority, 439 (64.4%), of the study participants reported they spend 30 minutes to go back and forth to school. More than sixty percent, 424 (62.2%), of the study participants had no illness in the past two weeks (Table 3).

### 3.4. Anthropometric Findings of the Participants

**Figure 1:**
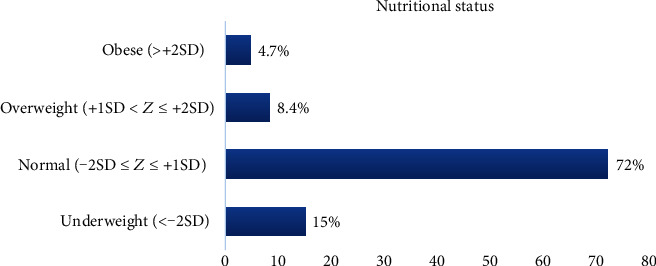
Nutritional status of adolescent school girls in Bahir Dar City, Northwest Ethiopia (*n* = 682).

One hundred two (15%), 57 (8.4%), and 32 (4.7%) of secondary school adolescent girls were found to be underweight, overweight, and obese, respectively (Figure 1).

**Figure 2:**
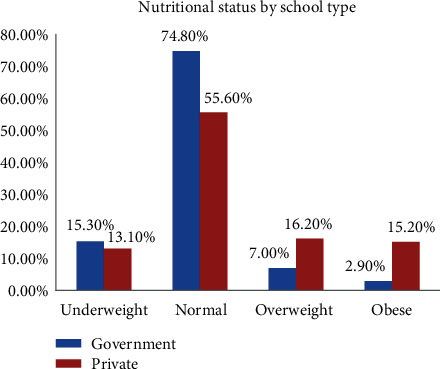
Nutritional status of adolescent school girls by school type in Bahir Dar City, Northwest Ethiopia.

In this study, high prevalence of overweight and obesity was found among private schools than among government schools **(**Figure 2).

### 3.5. Factors Associated with Underweight among Female School Adolescents

This study revealed that ages of the respondents, family size, meat intake per week, and onset of menarche were found to be associated with underweight. Adolescent age found between 14 and 16.5 years old were 1.7 times more likely to be underweight compared with counterparts (AOR: 1.7, 95% CI: 1.03-2.69). Respondents living with a family size of 4-6 were 2.8 times more likely to be underweight than those having a family size of ≤3 (AOR: 2.8, 95% CI: 1.05-4.99), whereas participants who did not eat meat once a week were1.6 times more likely to be underweight than those who ate meat two or more times per week (AOR: 1.6, 95% CI: 1.90-2.82). Moreover, girls with no onset of menarche were 4 times more likely to be underweight than the counterparts (AOR = 4.4, 95% CI: 1.21, 15.75 (Table 4).

N.B.: ^a^significant in the bivariable analysis; ^b^significant in the multivariable analysis; Ref: reference.

### 3.6. Factors Affecting Female School Adolescents' Overweight

Monthly family income, meat intake per week, and fruit intake per week were found to be independently associated with overweight. Adolescents whose monthly family income was above 6500 Ethiopian birr were 12.7 times more likely to be overweight than adolescents having family monthly income below 2500 Ethiopian birr (AOR: 12.7, 95% CI: 2.47-65.62). Study participants who ate meat two times and more per week were 2.1 times more likely to be overweight than the counterparts (AOR: 2.07, 95% CI: 1.47-9.14), whereas the risk of being overweight was found to be low among adolescents who ate fruits at least once per week compared with those who did not eat fruit (AOR: 0.20, 95% CI: 0.05-0.78) (Table 5).

N.B.: ^a^significant in the bivariable analysis; ^b^significant in the multivariable analysis; Ref: reference.

## 4. Discussion

Adolescent malnutrition both overweight and underweight is the major global health challenge of the 21^st^ century [21]. Adolescent girls are the most vulnerable group of the population, in which addressing the nutritional problems of this portion of the population is critical to save the future generations [22]. This study showed that adolescent girls were affected with the twin burden of malnutrition. In the current study, the prevalence of underweight was 102 (15%). The finding is found to be consistent with studies conducted in Poland [23] and Saudi Arabia [24]. However, the finding is lower than studies conducted in Sudan [25], India [26], and Bangladesh [27]. The difference could be due to the level of socioeconomic status (the aforementioned studies were conducted in rural and semiurban areas), the contrasting level of awareness of the community, and the study period.

In the current study, 54 (7.9%) adolescent girls face hunger at least once per month, a third of the girls did not eat meat at least once per week, around a quarter of female adolescents did not eat fruit per week, and only a quarter of them eats four and more meals per day. Despite this population group having rapid physical growth and the greatest nutritional need, the feeding practice was found to be poor. Inadequate nutritional intake in this population group might affect educational status, negatively affect physical growth, increase the risk of poor obstetric outcomes for teen mothers, jeopardize the healthy development of future children, and hurt future productivity and income generation potentials [21, 22]. Therefore, a design evidence-based female adolescent nutrition intervention shall be emphasized by the government and other concerned bodies to avert the effect of lifelong consequences of malnutrition.

Consistent with other studies [5, 11, 28–30], those in the early adolescent age group were 1.7 times more likely to be underweight than the counterparts. This might be related to the high nutrition demand of the body since it is a period of a growth spurt including physical, emotional, psychological, and sexual. On the contrary, in the late adolescent period, they might search food stuffs by themselves and reach maturation [22]. Therefore, avoiding constant dietary practice from childhood to early adolescent is very important, since the demand of food in amount and item in different age groups is different. Thus, creating awareness on adolescent feeding practice in the community shall be considered.

In line with studies [31, 32], those adolescents with late onset of menarche were 4 times more likely to be underweight than the counterparts. This might be due to girls needing adequate lean body fat and reaching a certain weight for menarche to occur. Studies showed that age at menarche is significantly associated with BMI and height of the girl. Those who have higher BMI are more likely to have menarche at lower ages [32–34]. On the other hand, those who are thin are more likely to have delayed menarche [32–34]. Similarly, those with short stature are likely to have delayed menarche [35]. Therefore, late onset of menarche might be a sign of undernutrition.

Adolescent girls living with more than four family members were 2.8 times more likely to be underweight than those having less than three. The finding is comparable with other studies [28, 36, 37]. This might be due to the presence of a large family size who might be sharing the available food stuffs. In addition, the finding which is explained by large family size is associated with poor economic and educational status [38].

In the present study, the prevalence of overweight and obesity was 8.4% and 4.7%, respectively. The finding is in line with studies conducted in Poland [23], Sudan [25], regional city of Nigeria [39], Addis Ababa (Ethiopia) [40], and India [41]. However, the finding is lower than a study conducted in Saudi Arabia [24] and higher than studies conducted in West Hararghe, Ethiopia [11], and Adama, Central Ethiopia [13]. The difference could be attributed to the variation in socioeconomic condition of the household and energy-related behavior including behavior of sedentary, physical activities, and eating behavior. Overnutrition in adolescent girls could predispose to different chronic medical illnesses such as cardiovascular, endocrine, gastrointestinal, neurological, psychosocial, and mental health problems [42–44]. Therefore, attention shall be given to overweight adolescents through implementing different strategies like creating awareness among the community on healthy feeding practice and physical activities and reducing sedentary life style. In addition, implementing a school-based nutrition program and screening adolescents for malnutrition shall be emphasized [45].

Participants who ate fruit at least once per week were less likely to be overweight. The finding is comparable with previous studies [46–50]. Therefore, encouraging fruit and vegetable intake is very important to reduce excessive weight gain. On the contrary, in this study, high meat intake per week was associated with weight again in a multivariable model. Those who ate meat more than two times per week were 2 times more likely to be overweight than the counterparts. The finding is in line with different previous studies [51–54]. This might be due to the fact that meat is one of the high energy dense foods and has high saturated fats [55].

Those with monthly family income more than 6500 Ethiopian birr were 12 times more likely to be overweight than those whose monthly income was below 2500 Ethiopian birr. The finding is consistent with different studies conducted in developing countries [56–59]. This could be due to those who had high monthly income in developing countries being more likely to take processed, westernized foods and use a vehicle for transport which are unhealthy practices. On the other hand, studies conducted in developed countries showed that poor socioeconomic status is associated with overweight [60–62].

This study shall be seen with the following limitations: First, using a cross-sectional study design might affect causal inference. Second, a dietary diversity score was not assessed. Third, we did not use any quantification for dietary assessment.

## 5. Conclusion

The prevalence of underweight and overweight was found to be high. Age of respondents, family size, meat intake per week, and onset of menarche were found to be associated with underweight, whereas monthly family income, meat intake, and fruit intake were found to be predictors of overweight. Designing an evidence-based nutritional intervention shall be emphasized by the government and other stakeholders to avert the dual burden of malnutrition.

## Figures and Tables

**Table 1:** Socioeconomic and demographic profile of adolescent school girls in Bahir Dar City, Northwest Ethiopia (*n* = 682).

Characteristics	Frequency	Percent
Age of the respondents		
14–16.5	231	39.9
16.6–19	451	66.1
Type of school		
Government	583	85.5
Private	99	14.5
Level of grade		
9^th^	248	36.4
10^th^	134	19.6
11^th^	125	18.3
12^th^	175	25.7
Marital status of the respondents		
Married	59	8.7
Single	617	90.5
Separated	6	8.8
Family size		
≤3	101	14.8
4-6	432	63.3
≥7	149	21.8
Religion		
Orthodox	543	79.6
Protestant	35	5.1
Muslim	101	14.8
Others	3	0.4
Ethnicity		
Amhara	668	97.9
Tigray	8	1.2
Others	6	0.9
Sex of head of the household		
Male	556	81.5
Female	126	18.5
Occupational status of the father		
Farmer	89	15.3
Government employee	298	43.7
Private employee (NGO, merchant, self-employed)	260	44.8
Others	29	4.3
Occupational status of the mother		
Farmer	44	6.5
Government employee	91	13.3
Private employee (NGO, merchant, self-employed)	167	24.5
Housewife	358	52.5
Others	22	3.2
Educational level of the father		
Illiterate	82	12.1
Primary school	166	24.5
Secondary school	136	20.1
Above secondary school	292	42.8
Educational level of the mother		
Illiterate	158	23.2
Primary school	197	28.9
Secondary school	180	26.4
Above secondary school	147	21.5
Monthly family income(Ethiopian birr)		
≤2500	187	27.4
2500-3999	125	18.3
4000-6000	203	29.8
≥6001	167	24.5
Vehicle use to come & back to school		
Yes	226	33.1
No	456	66.9

**Table 2:** Nutrition- and diet-related characteristics of female school adolescents in Bahir Dar City, Northwest Ethiopia (*n* = 682).

Characteristics	Frequency	Percent
Staple diet		
Teff	667	97.8
Wheat	2	0.3
Maize	4	0.6
Sorghum	2	0.3
Others	7	1.0
Meals per day		
One times	6	0.9
Two times	72	10.6
Three times	432	63.3
Four times and above	172	25.2
Vegetable intake per day		
No intake	89	13
One times	358	52.5
Two times	188	27.6
Three times and above	47	6.9
Fruit intake per week		
No intake	158	23.2
One times	344	50.4
Two times and above	180	26.4
Meat intake per week		
No intake	215	31.5
One times	158	23.2
Two times and above	309	45.3
Snack intake per day		
No intake	387	56.7
One times	239	35.1
Two times	56	8.2
Items taken for snack		
Cereal & other grains	165	55.7
Fruit and vegetable	129	43.6
Meat, egg, and milk	2	0.7
Hunger in the last 30 days due to shortage of food		
No hunger	585	85.8
One time	54	7.9
Two times	28	4.1
Three times	8	1.2
Four times and above	7	1.0
Additional food bought (consumed)		
Cake	126	18.5
Biscuit	310	45.5
Ice cream	57	8.4
Chocolate	159	23.3
Others	30	4.4

**Table 3:** Behavioral and life styles of female adolescents in Bahir Dar City, Northwest Ethiopia (*n* = 682).

Variables	Frequency	Percent
Hard physical activities per week		
None	414	60.7
One day	110	16.1
Two days	66	9.7
Three days	31	4.5
Four days and above	61	8.9
Moderate activities		
Yes	385	56.5
No	297	43.5
Sport done (gymnastic, swimming)		
Yes	188	27.6
No	494	72.4
Time taken to school		
≤15 min	77	11.3
16-30 min	439	64.4
31-60 min	144	21.1
≥60 min	22	3.2
Do you drink alcohol		
Yes	45	6.6
No	637	93.4
Did you smoke cigarette		
Yes	1	0.1
No	681	99.9
Did you have a latrine		
Yes	671	98.4
No	11	1.6
Did you have any illness in the past 2 weeks		
Yes	258	37.8
No	424	62.2
Sources of drinking water		
Tap water	676	99.1
Protected well	6	0.9

**Table 4:** Factors associated with underweight among female school adolescents in Bahir Dar City, Northwest Ethiopia (*n* = 682).

Characteristics	Underweight		COR (95% CI)	AOR (95% CI)
	Yes (%)	No (%)		
Ages of the respondents				
14–16.5	45 (6.6)	186 (27.3)	1.67 (1.1, 2.57)^a^	1.66 (1.03, 2.69)^b^
16.6–19	57 (8.4)	394 (57.8)	Ref	Ref
Family size				
≤3	10 (1.5)	91 (13.3)	Ref	Ref
4–6	72 (10.6)	360 (52.8)	1.82 (0.90, 3.70)	2.83 (1.05, 4.99)^b^
≥7	20 (2.9)	129 (18.9)	1.4 (0.60, 3.20)	1.60 (0.64, 4.29
Meat intake per week				
No intake	50 (7.3)	165 (24.2)	2.09 (1.32, 3.33)^a^	1.55 (1.90, 2.82)^b^
One time	13 (1.9)	145 (21.3)	0.62 (0.32, 1.20)	0.52 (0.26, 1.05)
Two times and above	39 (5.7)	270 (39.6)	Ref	Ref
Family income per month in ETB birr				
≤2500	39 (5.7)	148 (21.7)	1.83 (1.03, 3.26)^a^	1.13 (0.55, 2.31)
2500-3999	22 (3.2)	103 (15.1)	1.49 (0.78, 2.84)	1.2 (0.58, 2.49)
4000-6000	20 (2.9)	183 (26.8)	0.76 (0.40, 1.46)	0.67 (0.33, 1.36)
≥6001	21 (3.1)	146 (21.4)	Ref	Ref
Additional food bought (consumed)				
Cake	12 (1.8)	108 (15.8)	Ref	Ref
Biscuit	64 (9.4)	239 (35.0)	2.83 (1.34, 5.73)^a^	2.5 (0.30, 20.8)
Ice cream	5 (0.7)	45 (6.6)	0.97 (0.29, 3.27)	0.8 (0.70, 8.70)
Chocolate	17 (2.5)	136 (19.9)	1.27 (0.55, 2.91)	1.15 (0.13, 10.37)
Others (not much sweets)	9 (1.3)	47 (6.9)	1.86 (0.69, 5.02)	1.59 (0.17, 15.29)
Do you start menarche				
Yes	217 (31.8)	353 (51.8)	Ref	Ref
No	44 (6.5)	68 (10.0)	4.22 (1.31, 13.56)^a^	4.36 (1.21, 15.75)^b^

**Table 5:** Factors associated with overweight/obesity among female school adolescents in Bahir Dar City, Northwest Ethiopia (*n* = 682).

Characteristics	Over weight		COR (95% CI)	AOR (95% CI)
	Yes (%)	No (%)		
Meat intake per week				
No intake	17 (2.5)	198 (29.0)	Ref	Ref
One time	18 (2.6)	140 (20.5)	1.50 (0.75, 3.01)	4.31 (0.97, 19.27)
Two times and above	58 (8.5)	255 (37.4)	2.47 (1.39, 4.39)^a^	2.07 (1.47, 9.14)^b^
Fruit intake per week				
No intake	17 (2.5)	141 (20.7)	Ref	Ref
One time	39 (5.7)	305 (44.7)	1.06 (0.58, 1.94)	0.22 (0.06, 0.76)^b^
Two times and above	33 (4.8)	147 (21.6)	1.86 (0.99, 3.49)	0.2 (0.05, 0.78)^b^
Monthly family income in ETB birr				
≤2500	11 (1.6)	176 (25.8)	Ref	Ref
2500-3999	9 (1.3)	116 (17.0)	1.24 (0.50, 3.09)	2.48 (0.45, 13.83)
4000-6000	25 (3.7)	178 (26.1)	2.25 (1.07, 4.71)^a^	3.15 (0.62, 15.96)
≥6001	44 (6.5)	123 (18.0)	5.72 (2.84, 11.52)^a^	12.74 (2.47, 65.62)^b^
Use vehicle to travel to school				
Yes	40 (5.9)	186 (27.3)	1.79 (1.14, 2.81)^a^	0.86 (0.33, 2.25)
No	49 (7.2)	407 (59.7)	Ref	Ref

## Data Availability

The datasets used in this study are available from the corresponding author and can be accessed through request.

## Abbreviations

BMI: Body mass index
CI: Confidence interval
OR: Odds ratio
SD: Standard deviation.
